# Co-transplantation of Epidermal Neural Crest Stem Cells and Olfactory Ensheathing Cells Repairs Sciatic Nerve Defects in Rats

**DOI:** 10.3389/fncel.2019.00253

**Published:** 2019-06-12

**Authors:** Lu Zhang, Bingcang Li, Bin Liu, Zhifang Dong

**Affiliations:** ^1^Department of Pediatric Research Institute, Children’s Hospital of Chongqing Medical University, Ministry of Education Key Laboratory of Child Development and Disorders, China International Science and Technology Cooperation Base of Child Development and Critical Disorders, Chongqing Key Laboratory of Translational Medical Research in Cognitive Development and Learning and Memory Disorders, Chongqing, China; ^2^State Key Laboratory of Trauma, Burns and Combined Injury, Daping Hospital, Research Institute of Surgery, Third Military Medical University, Chongqing, China; ^3^Key Laboratory of Freshwater Fish Reproduction and Development, Ministry of Education, School of Life Sciences, Southwest University, Chongqing, China

**Keywords:** peripheral nerve injury, epidermal neural crest stem cell, olfactory ensheathing cell, co-transplantation, brain derived growth factor, nerve growth factor

## Abstract

Cell-based therapy is an alternative strategy to improve outcomes of peripheral nerve injury (PNI). Epidermal neural crest stem cell (EPI-NCSC) is obtained from autologous tissue without immunological rejection, which could expand quickly *in vitro* and is suitable candidate for cell-based therapy. Olfactory ensheathing cell (OEC) could secrete multiple neurotrophic factors (NTFs), which is often used to repair PNI individually. However, whether the combination of EPI-NCSC and OEC have better effects on PNI repair remains unclear. Here we use EPI-NCSC and OEC co-transplantation in a rat sciatic nerve defect model to ascertain the effects and potential mechanisms of cells co-transplantation on PNI. The effect of EPI-NCSC and OEC co-transplantation on PNI is assessed by using a combination of immunohistochemistry (IHC), electrophysiological recording and neural function test. Co-transplantation of EPI-NCSC and OEC exerts a beneficial effect upon PNI such as better organized structure, nerve function recovery, and lower motoneuron apoptosis. IHC and enzyme-linked immuno sorbent assay (ELISA) further demonstrate that cells co-transplantation may improve PNI via the expression of brain derived growth factor (BDNF) and nerve growth factor (NGF) up-regulated by EPI-NCSC and OEC synergistically. Eventually, the results from this study reveal that EPI-NCSC and OEC co-transplantation effectively repairs PNI through enhancing the level of BDNF and NGF, indicating that cells co-transplantation may serve as a fruitful avenue for PNI in clinic treatment.

## Introduction

Peripheral nerve injury continues to be a major challenge in reconstructive neurosurgery. Owing to huge clinical demand, peripheral nerve regeneration, particularly larger gap injuries, has become a prime focus of basic and clinical research. Accelerating axonal regeneration to promote reinnervation and improve functional recovery after PNI is a clinical necessity and an experimental challenge (Goel et al., 2009; Cotter et al., 2010; Tang et al., 2013).

For surgical repair of PNI with substantial neural defects, the current gold standard is to bridge the defect with an autologous nerve graft that is obtained from another part of the body. In terms of inevitable drawbacks associated with autologous nerve graft, the development of artificial substitutes for autologous nerve grafts is an urgent need in the field of regenerative medicine. NTE has been shown to satisfy this need. It is typically comprised of a neural scaffold with incorporated biochemical cues. The scaffold is prepared with a variety of synthetic or natural biomaterials through well-defined fabrication techniques. Among a large body of scaffold biomaterials, Poly(lactic-co-glycolic acid) (PLGA) (Bini et al., 2004), comprised of Polylactide (PLA) and Polyglycolide (PGA), shows excellent neural affinity and biocompatibility with cells. PLGA not only can be used to control the release of inosine, NGF and insulin-like growth factor (IGF), but also combine to seed cells such as Schwann cells, bone marrow stromal cells (BMSCs) or neural stem cells (NSCs), or can be processed into a graft for guiding peripheral nerve regeneration.

Seed cells, as an important component of NTE, provide an optional strategy for NTE with an enhanced ability to repair PNI. Attractively, EPI-NCSCs, combined with the advantage of adult stem cell and embryonic stem cell, represent a unusual type of multipotent adult stem cell and is a suitable candidate for cell-based therapy (Hu et al., 2006; Sieber-Blum et al., 2006). Accumulating evidences (Amoh et al., 2005) suggest that EPI-NCSCs can be induced to differentiate into Schwann cells and might secrete growth factors to modulate the behavior of Schwann cells. Our previous reports have showed that EPI-NCSCs could reduce inflammation (Li et al., 2017) and promote the segmental recovery of PNI (Zhang et al., 2014). Moreover, OECs are specialized glial cells somewhat similar to Schwann cells and astrocytes, which secrete NGF and BDNF, suggesting that NTFs produced by OECs might enhance the survival of damaged axons (Marshall et al., 2006). Indeed, abundant studies have reported that OECs are able to promote axonal regeneration and remyelination after SCI (Gomes et al., 2018; Gómez et al., 2018; Wright et al., 2018) and PNI (Radtke et al., 2009; Guérout et al., 2011). Importantly, the synergic effects of EPI-NCSCs and OECs improved locomotor function of contused spinal cord of rats and enhanced the expression of NTFs (Zhang et al., 2015). Based on these, the combination of EPI-NCSCs and OECs may synergistically repair PNI. Therefore, we will probe the mechanism that co-transplantation of EPI-NCSCs and OECs have reparative effects on PNI.

In the present study, we engineered nerve grafts by incorporating EPI-NCSCs and OECs as seed cells into PLGA to bridge a 10 mm long sciatic nerve defect in rats, and investigated therapeutic effects of cells co-transplantation by using IHC, electrophysiological recording *in vivo* and neural function test.

## Materials and Methods

### The Acquisition and Identification of EPI-NCSCs and OECs

#### Isolation and Culture of EPI-NCSCs and OECs

##### EPI-NCSCs

Epidermal neural crest stem cells were isolated and cultured following procedures described by Sieber-Blum et al. (2006) and Clewes et al. (2011). Sprague Dawley rats were anaesthetized with 3% pentobarbital sodium (Sigma, United States) (70 mg/kg), cut whisker pads, stripped hair follicle, adhered to collagen coated dish, added culture medium, and emigrated from expants on the third day. The protocol is described in the Supporting Information (Supplementary Material).

##### OECs

Olfactory ensheathing cells were isolated from adult green fluorescent protein (GFP)-Sprague Dawley rats by modification of the method described by Sasaki et al. (2004). Sprague Dawley rats were anaesthetized with 3% pentobarbital sodium (Sigma, United States) (70 mg/kg), separated olfactory bulbs, reserved outer nerve layer, minced tissue, digested with 0.25% trypsin for 30 min at 37°C, transferred to culture dish, and grew up on the 7th day. The protocol is described in the Supporting Information (Supplementary Material).

##### Identification of EPI-NCSCs and OECs

Washed cells with 0.01 M phosphate buffer (PBS) for three times, fixed with 4% Polyoxymethylene (PFA) for 30 min, permeated with 0.3% Triton X-100 for 30 min, blocked with 1% BSA for 30 min, incubated with primary antibody overnight at 4°C, washed with PBS for three times (5 min each time), then incubated with second antibody for 1 h at RT, washed with PBS for three times (10 min each time), dried at RT, covered with Fluoromount anti-fade reagent (Sigma, United States), and observed with fluorescence microscope. The protocol is described in the Supporting Information (Supplementary Material).

##### Fabrication and degradation of nerve conduits

Nerve conduits were fabricated and degraded following procedures described by Moore et al. (2006) and Li et al. (2010). The nerve conduits were fabricated from 10% PLGA dissolved in CHCl_3_ (Sigma, United States), cut into 15 mm length, and sterilized by gamma irradiation for 30 min. The nerve conduits were put in heat-sealed pouch, vacuum-dried for 24 h to obtain tare weights. Then, samples were put in 0.01 M PBS, and incubated at 37°C in 5% CO_2_ incubator. At regular intervals, monitored pH and weighed. Details are provided in the Supporting Information (Supplementary Material).

##### Animal model and transplantation

Fifty Sprague-Dawley rats (Laboratory Animal Center, Third Military Medical University, Chongqing, China) weighing 220–250 g were used in all groups. Experiment is divided into five groups: (1) DMEM/F_12_ (*n* = 10); (2) EPI-NCSC (*n* = 10); (3) OEC (*n* = 10); (4) EPI-NCSC+OEC (*n* = 10); (5) Control (*n* = 10). The right sciatic nerve as experimental side, the left sciatic nerve or normal animals as control. All experimental procedures with animals were approved by the local institution review board and were carried out according to the guidelines of the Third Military Medical University (Chongqing, China) for the care and use of laboratory animals. Details are provided in the Supporting Information (Supplementary Material).

##### Histological observation and survival of transplanted cells *in vivo*

The H&E staining protocol was used to assess organization of structures. The graft was harvested and fixed with 4% PFA 8 weeks after transplantation. Samples derived from the middle portion of the graft were longitudinally sectioned into 20 μm thickness section for H&E staining. Details are provided in the Supporting Information (Supplementary Material).

### Electrophysiology and Retrograde Tracing

#### Electrophysiology

To evaluate nerve regeneration, *in vivo* electrophysiology was performed. Stimuli electrodes (strength: 3 mA; interval: 0.25 ms) were placed under the sciatic nerve trunk 5 mm proximal to the suturing point and recording electrodes were inserted in the gastrocnemius. cMAPs was gathered by data acquisition software (Power lab, Australia). NCV was calculated by recording latency time of proximal and distal end. Normal nerve as control. Eight weeks after transplantation, five rats in each group were randomly chosen for electrophysiology.

#### Retrograde Tracing

Retrograde tracing was used to assess nerve regeneration. Eight weeks after transplantation, the sciatic nerve was exposed under anesthetization, and 2% DiI solution (15 μL) was injected into the nerve trunk 10 mm proximal to the suturing point with a microinjector. After the injection, the needle was kept *in situ* for 5 min. After 24 h, gathered samples, sectioned transversely into 15 μm sections, and observed by fluorescence microscope (BX 51WI, Olympus). Details are provided in the Supporting Information (Supplementary Material).

#### Analysis of the Expression of BDNF and NGF

Brain derived growth factor and NGF play a vital role in repairing PNI, therefore we test the expression of BDNF and NGF by IHC and ELISA eight after transplantation. The rats were anesthetized with 3% pentobarbital sodium (70 mg/kg). Samples were harvested and sectioned into 15 μm sections. Incubated with primary antibody overnight at 4°C, then incubated with second antibody for 1 h at 37°C, and washed three times with PBST. Samples were observed by fluorescence microscope (BX 51WI, Olympus).

Eight weeks after transplantation, the expression of BDNF and NGF was quantified by ELISA. Samples were harvested. The concentration of BDNF and NGF was measured by ELISA kit (Abcam, United States) according to the manufacturer’s instructions. Details are provided in the Supporting Information (Supplementary Material).

#### Evaluation of Myelination and Cell Apoptosis

Transmission electron microscopy and toluidine blue staining were used to evaluated the myelination and cell apoptosis was assessed by TUNEL staining. Eight weeks after transplantation, the graft was harvested, cut into ultrathin sections to stain with lead citrate and uranyl acetate, and followed by observation under transmission electron microscope. TUNEL staining was carried out by *in situ* Cell Death Detection Kit (Roche, Germany) according to the manufacturer’s instructions. Details are provided in the Supporting Information (Supplementary Material).

### Behavior Test and Gastrocnemius Assessment

#### Sciatic Function Index

The evaluation of locomotor function was performed by the SFI according to methods described by Bain et al. (1989). Rats were acclimatized experiments before surgery. Foot prints from the normal (N, right side) and experimental (E, left side) were recorded 2, 4, and 8 weeks after transplantation. SFI ranged from -100 to 0. The value 0 describes normal function and the value -100 shows complete transaction of sciatic nerve. Details are provided in the Supporting Information (Supplementary Material).

#### Withdrawal Latency

The assessment of sensorial function was carried out by hot bath (Hargreaves et al., 1988; Alleva et al., 1997; Inoue et al., 2004). The hind paw was immersed in 50 ± 1°C hot water bath to measure the withdrawal latency. Rats were acclimatized experiments before surgery. In test session, each rat was tested in five trials with an interval of 5 min. The hind paw withdrawal latencies were calculated as the mean of five trials. The maximum of withdrawal latency was set at 5 s to prevent tissue damage.

#### Gastrocnemius Assessment

Samples were harvested and weighed 2, 4, and 8 weeks after transplantation. The weight rate of the gastrocnemius (right/left) was used to assess nerve regeneration.

#### Statistical Analysis

All data were expressed as mean ± SD. All statistical analyses were carried out in SPSS17.0 software. Differences among groups were assessed by one-way ANOVA test. Two-way ANOVA test was used to analyze SFI and withdrawal latency in different groups and 2, 4, and 8 weeks after transplantation. One or two-way ANOVA tests were followed by the Bonferroni *post hoc* test. A *p*-value of <0.05 was set as the criteria for statistical significance.

## Results

### Characterization of EPI-NCSCs and OECs

**FIGURE 1:**
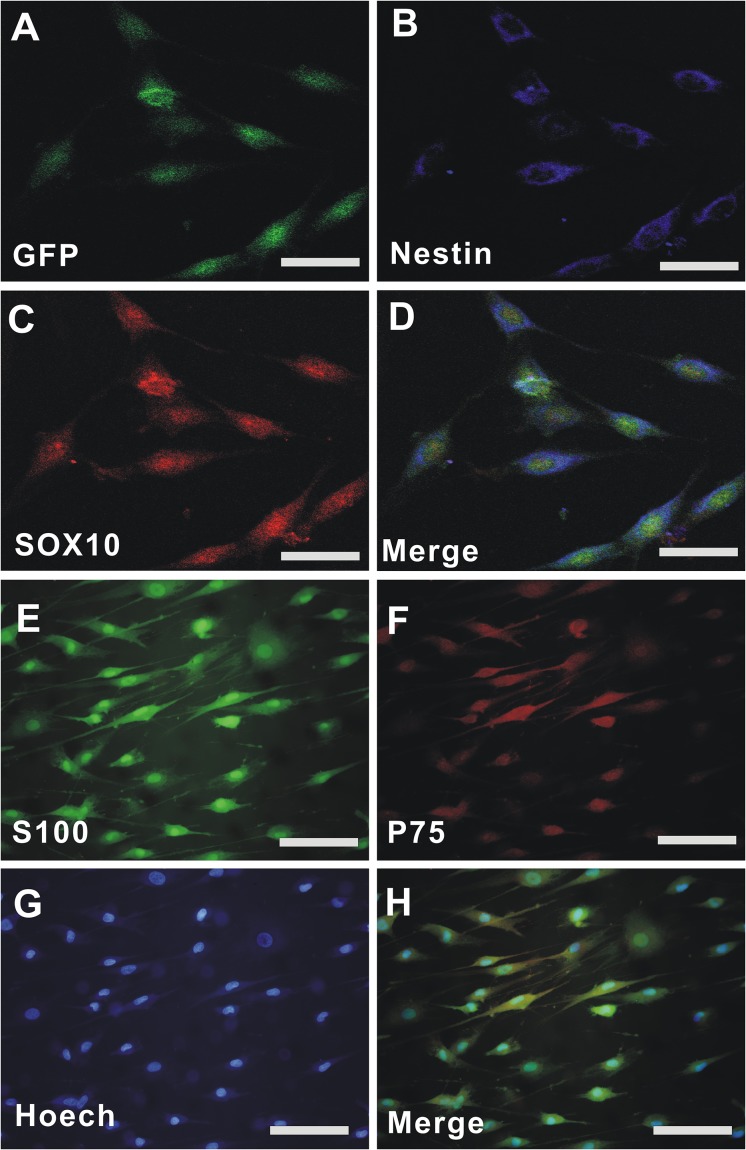
The culture and identification of EPI-NCSCs and OECs. **(A)** GFP-EPI-NCSC. **(B)** Nestin. **(C)** SOX10. **(D)** Merged GFP/Nestin/SOX10. **(E)** S-100. **(F)** p75. **(G)** Hoechst 33342. **(H)** Merged S-100/p75/Hoechst 33342. Nuclei were stained by Hoechst 33342 (blue). Scale for **A–D**, 15 μm; Scale for **E–H**, 25 μm.

Epidermal neural crest stem cells migrated from the bulge of hair follicles of GFP-rats and subcultured for 3 passages (P_3_), and the cells displayed a spindle-like shape (Green, Figure 1A) with green fluorescent protein (GFP). Double immunofluorescent staining demonstrated that the cells were positive for Nestin (Blue, Figure 1B), SOX10 (Red, Figure 1C), and Nestin/SOX10/GFP (Merged, Figure 1D). OECs were isolated from olfactory bulb of rats and cultured for P3. Immunofluorescent staining demonstrated that the cells were positive for S-100 (Green, Figure 1E), p75 (Red, Figure 1F), Hoechst 33342 (Blue, Figure 1G), and S-100/p75/ Hoechst 33342 (Merged, Figure 1H). The purity of EPI-NCSCs and OECs is above 95%.

### The Feature of the Nerve Conduits

**FIGURE 2:**
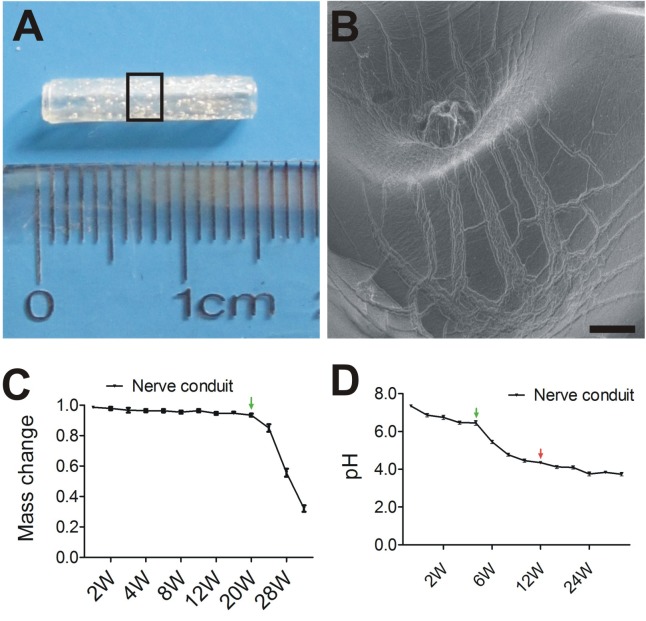
Tissue engineered nerve conduits for peripheral nerve regeneration. **(A)** The nerve conduit. **(B)** SEM of longitudinal sections of nerve conduit in enlarged rectangle box. **(C)** The profile of Mass change. **(D)** The profile of pH change. **B**, Scale = 100 μm; Values are shown as mean ± SD (*n* = 3).

To explore the potential therapeutic effects of EPI-NCSC and OEC co-transplantation on PNI, we constructed a 15-mm nerve conduit to repair the rat sciatic nerve defect (Figure 2A). The internal surface of nerve conduit was a little rough (Figure 2B) and had a few micropores for cell adhesion and migration.

The degradation of the nerve conduits under simulated physiologic conditions was estimated as expected for PLGA with the given copolymer ratio. As shown by the pH change curve in Figure 2D, pH decreased from 7.4 to 3.8 up to 28 weeks. Markedly, pH change from 4 weeks (green arrows) to 12 weeks (red arrows). In contrast, little or no mass loss was observed at 20 weeks (Figure 2C), followed by a period of more precipitous mass loss from 20 weeks (green arrows) to 34 weeks. The curve of mass change is typical for PLGA degradation.

### Histological Observation

**FIGURE 3:**
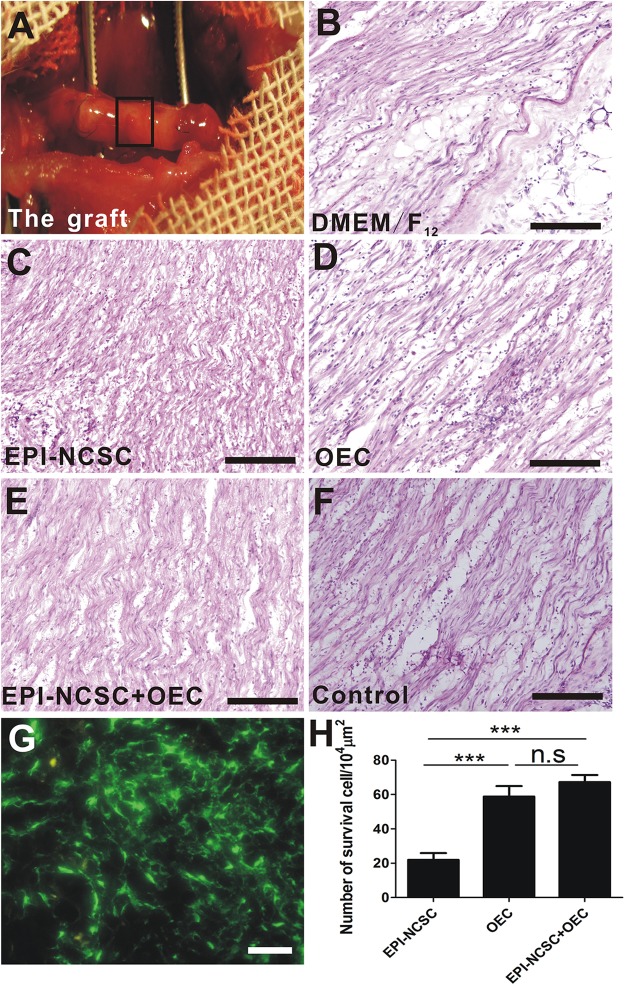
The histological observation of the graft and cell survival *in vivo* 8 weeks after transplantation. **(A)** The graft. HE staining of longitudinal sections from **(B)** the DMEM/F_12_ group, **(C)** the EPI-NCSC group, **(D)** the OEC group, **(E)** the EPI-NCSC+OEC group, **(F)** the Control group. **(G)** Transplanted GFP-cells, **(H)** the number of surviving cells. **(B–F)** Scale, 100 μm; **(G)** Scale, 15 μm; Values are shown as mean ± SD (*n* = 6); ^∗∗∗^*P* < 0.001; n.s., not significant.

Eight weeks after transplantation, the graft was exposed (Figure 3A). It was not degraded with intact shape and it distributed some blood vessels on the surface of the graft. H&E staining showed a sparse organized structure with few cells in vehicle group (DMEM/F_12_, Figure 3B), whereas the organized structure was compact and with greater cell numbers in cell transplantation groups (Figure 3C–E). The organized structure in control group (Figure 3F) was more regular than cell transplantation groups and DMEM/F_12_ group. Although EPI-NCSCs (Figure 3C) or OECs (Figure 3D) transplantation alone can integrate into the donor, co-transplantation of EPI-NCSCs and OECs (EPI-NCSC+OEC, Figure 3E) displayed better effects, as reflected by much more surviving cells in EPI-NCSC+OEC group compared to EPI-NCSCs or OECs transplantation alone (^∗∗∗^*P* < 0.001; Figure 3H). However, it showed that there is no significant difference of survival number between single transplantation of OEC and co-transplantation of EPI-NCSC and OEC, which indicated that transplantation microenvironment may be more suitable for OECs growth rather than EPI-NCSCs. These results indicate that transplanted cells are able to survive, migrate at the graft, and partly make up for nerve defects.

### EPI-NCSC and OEC Co-transplantation Promotes Nerve Regeneration

#### Electrophysiology

**FIGURE 4:**
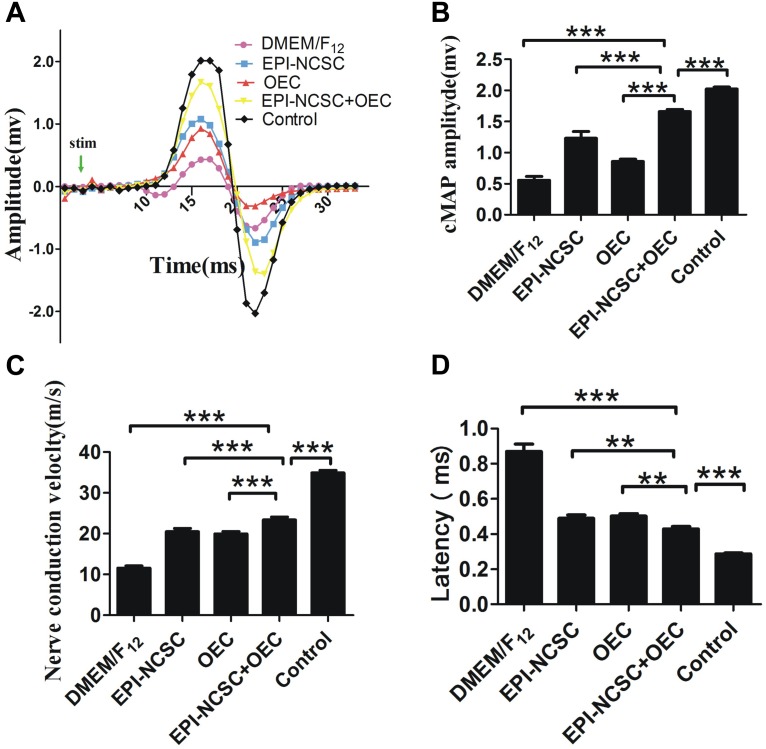
Examination of cMAPs and NCV 8 weeks after transplantation. **(A)** cMAPs traces of the DMEM/F_12_, EPI-NCSC, OEC, EPI-NCSC+OEC, and control groups. **(B)** Histogram shows latency in the different groups. **(C)** Histogram shows NCVs in the different groups. **(D)** Histogram shows cMAPs in the different groups. Values are shown as mean ± SD (*n* = 5); ^∗∗∗^*P* < 0.001, ^∗∗^*P* < 0.01.

Eight weeks after transplantation, cMAPs was recorded in the different groups (Figure 4A). The results showed that the amplitude of cMAPs was much bigger in the EPI-NCSC, OEC, and EPI-NCSC+OEC groups than that recorded in the DMEM/F_12_ group (^∗∗∗^*P* < 0.001; Figure 4B). Notably, although the amplitude of cMAPs in EPI-NCSC+OEC group was much bigger than that recorded in the EPI-NCSC or OEC group (^∗∗∗^*P* < 0.001, Figure 4B), it was still smaller than that recorded in the control group (^∗∗∗^*P* < 0.001, Figure 4B). The latency in the EPI-NCSC+OEC group was less than in the EPI-NCSC (^∗∗^*P* < 0.01, Figure 4D), OEC (^∗∗^*P* < 0.01, Figure 4D), and DMEM/F_12_ groups (^∗∗∗^*P* < 0.001, Figure 4D). Similarly, the NCVs in EPI-NCSC+OEC group was faster than that in the EPI-NCSC or OEC group (^∗∗∗^*P* < 0.001, Figure 4C). Taken together, these results indicate that co-transplantation of EPI-NCSCs and OECs improves nerve regeneration better than single EPI-NCSCs or single OECs.

#### Retrograde Tracing and Behavior Assessment

**FIGURE 5:**
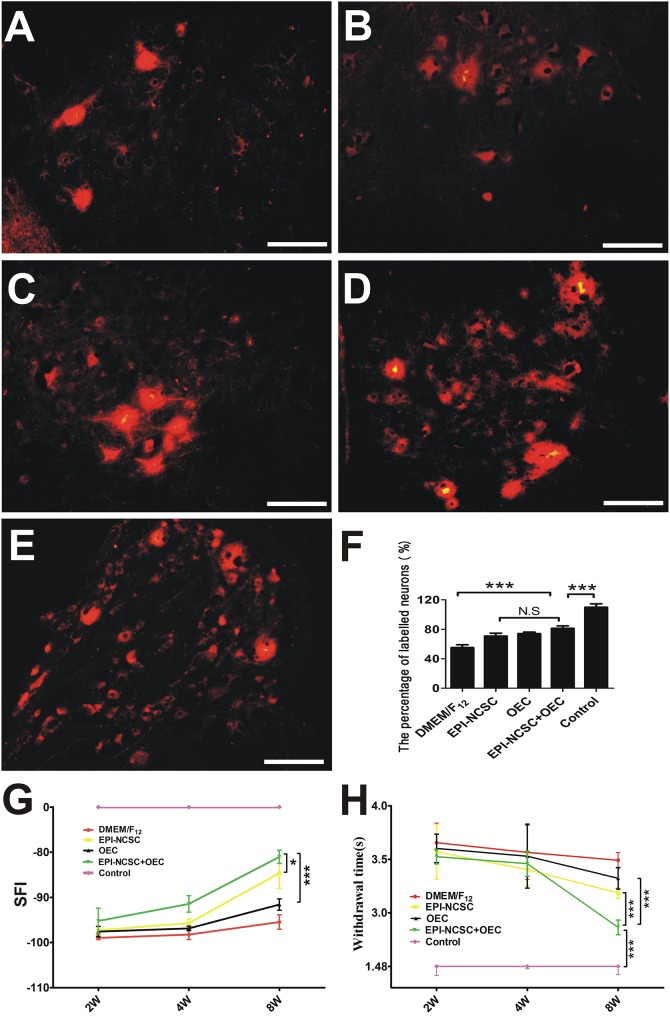
The assessment of nerve function 8 weeks after transplantation. DiI-labeled motoneurons of SCL_4-6_ in **(A)** the DMEM/F_12_ group, **(B)** the EPI-NCSC group, **(C)** the OEC group, **(D)** the EPI-NCSC+OEC group, **(E)** the Control group, **(F)** histogram showing the ratio of DiI-labeled motoneurons in all groups, **(G)** motor function assessment by SFI 2, 4, and 8 weeks after transplantation, **(H)** sensory function assessment by withdrawal time from a hot water bath 2, 4, and 8 weeks after transplantation. Scale, 15 μm, Values are shown as mean ± SD (*n* = 5); N.S: no significant difference, ^∗^*P* < 0.05, ^∗∗∗^*P* < 0.001.

DiI-labeled SCL_4-6_ motoneurons were examined 8 weeks after transplantation in DMEM/F_12_ (Figure 5A), EPI-NCSC (Figure 5B), OEC (Figure 5C), EPI-NCSC+OEC (Figure 5D) and control groups (Figure 5E). The ratio of DiI-labeled SCL_4-6_ motoneurons in the EPI-NCSC+OEC group was higher than that in the DMEM/F_12_ (^∗∗∗^*P* < 0.001), but lower than the Control group (^∗∗∗^*P* < 0.001, Figure 5F). The ratio of DiI-labeled SCL_4-6_ motoneurons in the EPI-NCSC+OEC group had no significant difference compared with the EPI-NCSC or OEC group (Figure 5F). Locomotor and sensorial functions were assessed by the SFI and limb withdrawal latency from a hot water bath at 2, 4, and 8 weeks after transplantation (Figure 5G,H). Although the SFI displayed no difference among these cell transplantation groups at 2 and 4 weeks, the SFI in the EPI-NCSC+OEC group was much bigger than that in the DMEM/F_12_ (^∗∗∗^*P* < 0.001, Figure 5G), OEC (^∗∗∗^*P* < 0.001, Figure 5G) and EPI-NCSC groups (^∗^*P* < 0.05, Figure 5G) at 8 weeks after transplantation. Notably, the SFI in the EPI-NCSC+OEC group was still lower than that in the control group (^∗∗∗^*P* < 0.001, Figure 5G). Similarly, the withdrawal latency in the EPI-NCSC+OEC group was shorter than that in the DMEM/F_12_ (^∗∗∗^*P* < 0.001, Figure 5H), EPI-NCSC (*P* < 0.001, Figure 5H) and OEC groups (^∗∗∗^*P* < 0.001, Figure 5H), but still shorter than that in the control group (^∗∗∗^*P* < 0.001, Figure 5H). Altogether, these behavioral results indicate that co-transplantation of EPI-NCSCs and OECs has better effect on improving motor and sensory functions in rats subjected to PNI.

#### Toluidine Blue Staining and TEM

**FIGURE 6:**
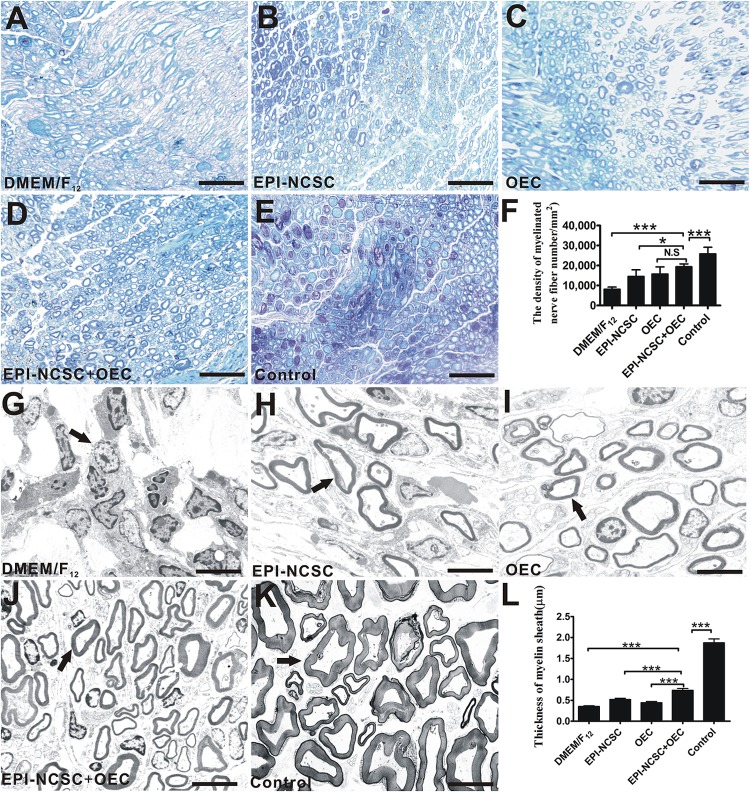
Toluidine blue staining and TEM of regenerated nerves 8 weeks after transplantation. Light microscopy images of toluidine blue staining of **(A)** the DMEM/F_12_ group. **(B)** The EPI-NCSC group. **(C)** The OEC group, **(D)** the EPI-NCSC+OEC group, and **(E)** the Control group. **(F)** Analysis of the density of myelinated nerve fibers. TEM images of ultrathin sections showing myelinated nerve fibers in **(G)** the DMEM/F_12_ group, **(H)** the EPI-NCSC group, **(I)** the OEC group, **(J)** the EPI-NCSC+OEC group, and **(K)** the Control group. **(L)** Analysis of the thickness of myelinated nerve fibers. **A–D**: Scale, 50 μm; **E–H**: Scale, 10 μm; Values are shown as mean ± SD (*n* = 5); N.S: no significant difference, ^∗^*P* < 0.05; ^∗∗∗^*P* < 0.001.

Eight weeks after transplantation, toluidine blue staining was performed to assess the remyelination of injured nerves. The results showed that the density of myelinated axons in the EPI-NCSC+OEC group was greater than that in the EPI-NCSC (^∗^*P* < 0.05, Figure 6F), and DMEM/F_12_ groups (^∗∗∗^*P* < 0.001, Figure 6F), but still less than that in the control group (^∗∗∗^*P* < 0.001, Figure 6F). In addition, the density of myelinated axons in the EPI-NCSC+OEC group has no significant difference vs. the OEC group. Further TEM analysis showed that the mean thickness of myelinated nerve fibers in the EPI-NCSC+OEC group was much bigger than that in the EPI-NCSC (^∗∗∗^*P* < 0.001, Figure 6L), OEC (^∗∗∗^*P* < 0.001, Figure 6L) and DMEM/F_12_ groups (^∗∗∗^*P* < 0.001, Figure 6L), but still less than that in the control group (^∗∗∗^*P* < 0.001, Figure 6L). In general, toluidine blue staining and TEM demonstrate that co-transplantation EPI-NCSC and OEC effectively facilitate the myelinated axons regeneration compared to single transplantation of EPI-NCSC or OEC.

#### TUNEL Staining and Gastrocnemius Recovery

**FIGURE 7:**
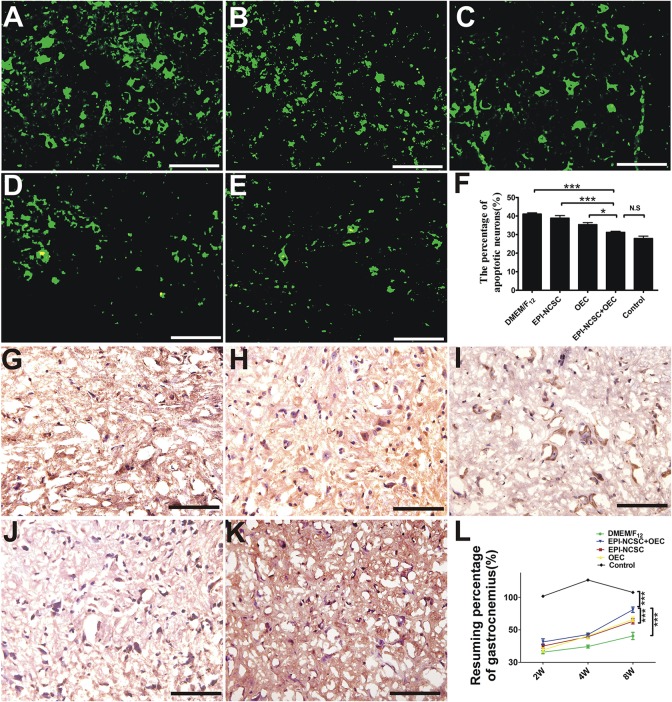
TUNEL staining and gastrocnemius recovery after transplantation. **(A)** Fluorescent image of motoneurons apoptosis in the DMEM/F_12_ group. **(B)** The EPI-NCSC group. **(C)** The OEC group. **(D)** The EPI-NCSC+OEC group. **(E)** The Control group. TNUEL staining of motoneurons apoptosis in **(F)** histogram of the percentage of apoptosis motoneuron. **(G)** The DMEM/F_12_ group. **(H)** The EPI-NCSC group. **(I)** The OEC group. **(J)** The EPI-NCSC+OEC group. **(K)** The Control group **(L)** the assessment of gastrocnemius 2, 4, and 8 weeks after transplantation. Scale = 25 μm; Values are shown as mean ± SD (*n* = 5); N.S: no significant difference, ^∗^*P* < 0.05, ^∗∗∗^ *P* < 0.001.

TUNEL staining displays that the percentage of motoneuron apoptosis in the EPI-NCSC+OEC group is lower than that in the DMEM/F_12_ (^∗∗∗^*P* < 0.001, Figure 7F), EPI-NCSC (^∗∗∗^*P* < 0.001, Figure 7F) and OEC groups (^∗^*P* < 0.05, Figure 7F), but has no significant difference compared to the control group (Figure 7F). Neurons with green fluorescence are apoptotic (Figure 7A–E), and puce cells are also apoptotic neurons (Figure 7G–K). The resuming ratio of gastrocnemius in the EPI-NCSC+OEC group is bigger than that in the DMEM/F_12_ group (^∗∗∗^*P* < 0.001, Figure 7L), EPI-NCSC (^∗∗∗^*P* < 0.001, Figure 7L), and OEC groups (^∗∗∗^*P* < 0.001, Figure 7L), but lower than in the control group (^∗∗∗^*P* < 0.001, Figure 7L) 8 weeks after transplantation. In conclusion, these results indicate that the co-transplantation of EPI-NCSCs and OECs decreases motoneuron apoptosis and improves gastrocnemius recovery after PNI.

#### Analysis of BDNF and NGF

**FIGURE 8:**
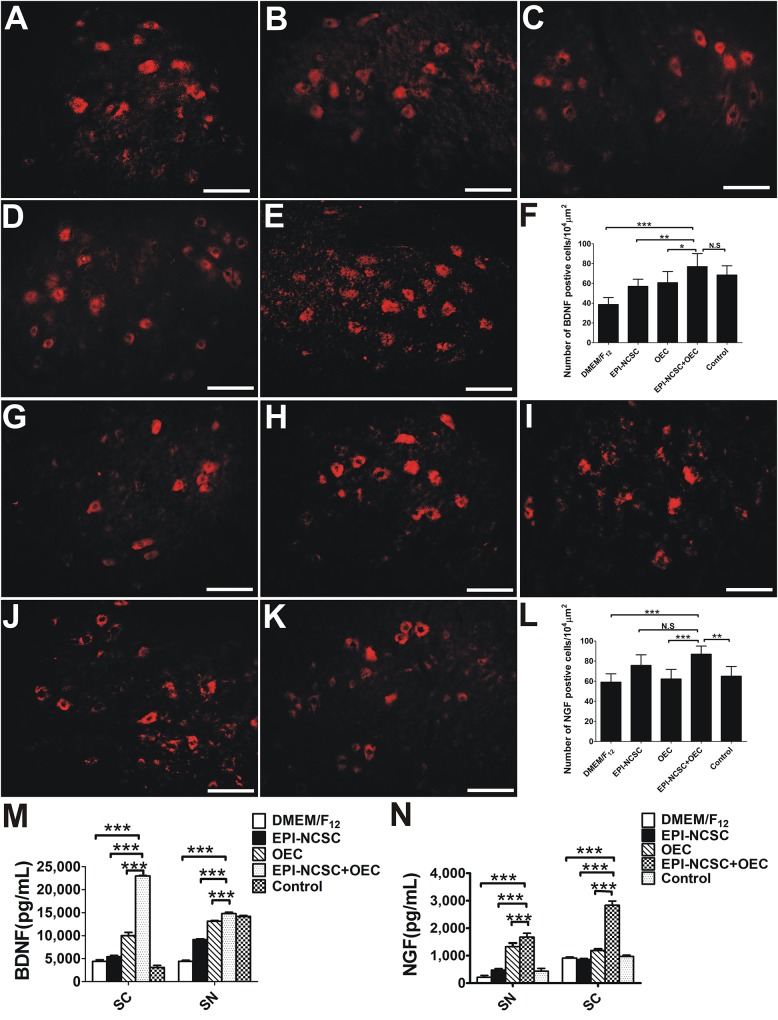
The expression analysis of BDNF and NGF 8 weeks after transplantation. Immunofluorescent staining of BDNF in **(A)** the DMEM/F_12_ group, **(B)** the EPI-NCSC group, **(C)** the OEC group, **(D)** the EPI-NCSC+OEC group, and **(E)** the Control group. **(F)** Histogram of the number of BDNF positive cells in each group. Immunofluorescent staining of NGF in **(G)** the DMEM/F_12_ group, **(H)** the EPI-NCSC group, **(I)** the OEC group, **(J)** the EPI-NCSC+OEC group, and **(K)** the Control group. **(L)** Histogram of the number of NGF positive cells in each group, **(M)** expression levels of BDNF in SC and SN, **(N)** expression levels of NGF in SC and SN. Scale, 20 μm; values are shown as mean ± SD (*n* = 5); N.S: no significant difference, ^∗^*P* < 0.05, ^∗∗^*P* < 0.01, ^∗∗∗^*P* < 0.001.

To elucidate the potential mechanisms by which co-transplantation of EPI-NCSCs and OECs promoted the regeneration of peripheral nerves, we next measured the levels of BDNF and NGF. IHC for BDNF showed that the number of BDNF positive cells in the EPI-NCSC+OEC group was greater than that in the EPI-NCSC (^∗∗^*P* < 0.01, Figure 8F), OEC (^∗^*P* < 0.05, Figure 8F), and DMEM/F_12_ groups (^∗∗∗^*P* < 0.001, Figure 8F), but has no significant difference compared to the control group (Figure 8F). The number of NGF positive cells in the EPI-NCSC+OEC group is greater than that in all other groups including the DMEM/F_12_ (^∗∗∗^*P* < 0.001, Figure 8L), OEC (^∗∗∗^*P* < 0.001, Figure 8L), and control groups (^∗∗^*P* < 0.01, Figure 8L), but has no significant difference compared to the EPI-NCSC group.

We next directly detected the expression level of BDNF and NGF both in the SN and in the SC 8 weeks after transplantation by ELISA. The results showed that the amount of BDNF in the SN and SC was much higher in the EPI-NCSC+OEC group, compared with the EPI-NCSC (^∗∗∗^*P* < 0.001, Figure 8M), OEC (^∗∗∗^*P* < 0.001, Figure 8M), and DMEM/F_12_ groups (^∗∗∗^*P* < 0.001, Figure 8M). Similarly, the amount of NGF in the SN and SC was also much higher in the EPI-NCSC+OEC group, compared with the EPI-NCSC (^∗∗∗^*P* < 0.001, Figure 8N), OEC (^∗∗∗^*P* < 0.001, Figure 8N) and DMEM/F_12_ groups (^∗∗∗^*P* < 0.001, Figure 8N).

In short, these results indicate that co-transplantation of EPI-NCSCs and OECs significantly increases the expression of BDNF and NGF, and then may consequently promote nerve regeneration.

## Discussion

The poor outcome after PNI has provoked researchers to improve methodologies for peripheral nerve regeneration (Xu et al., 2012; Hu et al., 2013). In this study, we used a rat sciatic nerve defect model to demonstrate that EPI-NCSC and OEC co-transplantation may ameliorate PNI. We further employed histology (TEM, IHC), electrophysiology *in vivo*, behavioral tests and retrograde tracing methods to identify the facilitation of peripheral nerve regeneration and nerve function recovery after co-transplantation.

Peripheral nerve system has a great regeneration potential, particularly when there is an appropriate microenvironment, such nerve conduits applied to a nerve defect that provide a guide and a biological environment for nerve regeneration. During the past few years, studies have been focused on various conduit materials, particularly biodegradable polymers such as poly(glycolic acid) (PGA) (Waitayawinyu et al., 2007), poly(L-lactic acid) (PLLA) (Hood et al., 2009), polycaprolactone (PCL) (Mligiliche et al., 2003) and Poly(lactic-co-glycolic acid) (PLGA) (Bini et al., 2004). These studies have indicated that the conduit itself does not have a pronounced effect on nerve repair. Thus, approaches to nerve repair are now focused on the optimization of the combination of nerve conduits such as NTFs (Tang et al., 2013), ECM (Li et al., 2010, 2017), and seed cells (Gu et al., 2014). We have reported that PLGA is strong (Figure 2C, 3A), and has neural affinity and biocompatibility with cells (Zhang et al., 2014), which are ideal graft properties for nerve regeneration (Li et al., 2010; Zhang et al., 2014). Interestingly, ECM provides well microenvironment, which regulate cell adhesion, spreading, and proliferation, and promote cell survival and myelination (Chernousov et al., 2008). Therefore, in this study transplanted cells combined with ECM may facilitate transplanted cell adhesion, spreading, proliferation, and cell survival. These are in line with the results in our study (Figure 3G,H).

Cell transplantation provides great potential for enhancement of nerve regeneration in view of NTFs, anti-inflammatory effect, as well as axon regeneration. EPI-NCSCs is a preferred candidate for cell transplantation, due to multipotent potential, autologous tissue without immune issue, and easy obtainment. What’s more, it combines the virtue of adult stem cells and embryonic stem cells. EPI-NCSCs, originated from the embryonic neural crest, can be easily obtained from the bulge of hair follicles without harmlessness for the donor (Figure 1A–D), which expand quickly *in vitro*. Recent studies have demonstrated the utilization of stem cells for peripheral nerve regeneration (Amoh et al., 2005). OECs secrete many NTFs including BDNF and NGF, which is often used to repair PNI (Guérout et al., 2011; Zhu et al., 2014; Ruiz-Mendoza et al., 2016; Wright et al., 2018). Based on the above, we co-implanted EPI-NCSCs and OECs into nerve conduits that were applied to a rat sciatic nerve defect model, demonstrating that EPI-NCSC and OEC co-transplantation increased the number of myelin sheathes (Figure 6D,I,K) and facilitated functional nerve recovery (Figure 5G,H).

The recovery of locomotor function is assessed by the SFI (de Medinaceli et al., 1982). In the present study, the SFI of the EPI-NCSC+OEC group was higher than that the DMEM/F_12_ group 8 weeks after transplantation (Figure 5G). This is consistent with report that transplanted OECs in a transected sciatic nerve model improved the motor function 3 weeks after surgery (Radtke et al., 2009). In the sensory function test, withdrawal time from hot water was employed to assess the recovery of sensory function (Röyttä et al., 1999). A previous study demonstrated that transplanted EPI-NCSCs promoted sensory recovery in response to warm water withdrawal (Li et al., 2017); we showed a similar but modest effect, whereas we observed a quicker withdrawal response in the EPI-NCSC+OEC group when compared with the other groups, except that the withdrawal response never came to reaching the control group (Figure 5H). This indicated that EPI-NCSC+OEC co-transplantation promoted the partial recovery of motor and sensory function. We further assessed the function of the regenerating sciatic nerves by electrophysiology *in vivo*. The results indicated that NCV, cMAPs and latency in the EPI-NCSC+OEC group were improved compared with the individual cell group (Figure 4). Previous studies have reported that transplanted neural stem cells improve NCV in a rat sciatic nerve transection model (Xu et al., 2012), that EPI-NCSCs promote the recovery of sciatic nerve cMAPs (Li et al., 2017), and that transplanted OECs improved sciatic nerve NCV and cMAPs in a rat sciatic nerve defect model (Li et al., 2010). We further performed gross observations and histological analyses by HE and TEM, which showed that the number, and thickness of myelin sheathes in the EPI-NCSC+OEC group were greater than in the individual cell group (Figure 6), similar to a previous study showing that transplanted OECs promoted the recovery of myelin sheathes in a rat sciatic nerve defect model (Franklin et al., 1996; Li et al., 2010). However, it reported that OECs was capable of remyelinating demyelinated CNS axons following transplantation into rat spinal cord injuries (Barnett et al., 2000; Kato et al., 2000) and promoted axon sprouting in the lesioned spinal cord (Richter et al., 2005), which indicated that OECs might play a crucial role in the regeneration of myelin sheathes and axon. To assess the accuracy of axon regeneration by EPI-NCSC+OEC co-transplantation, we used DiI retrograde tracing methods. The results indicated that the number of DiI-labeled motoneurons in SCL_4-6_ in the EPI-NCSC+OEC group was greater than in the DMEM/F_12_ group (Figure 5). Further, EPI-NCSC+OEC co-transplantation reduced motoneuron apoptosis in SCL_4-6_ 8 weeks after implantation (Figure 7), similar to a previous study using horseradish peroxidase retrograde tracing that demonstrated greater neuron survival following NCSCs transplantation than in the control group 52 weeks after transplantation (Lin et al., 2009). Strikingly, triple fluorescent retrograde tracing determined that OECs promoted the recovery of facial motor nerves by stimulating axonal sprouting (Guntinas-Lichius et al., 2001). Additionally, the resuming ratio of gastrocnemius in the EPI-NCSC+OEC group was higher than the EPI-NCSC, OEC, and DMEM/F_12_ groups (Figure 7L) which further indicated that EPI-NCSC+OEC co-transplantation promoted neurotrophic support to the recovery of gastrocnemius. Overall, our results indicate that EPI-NCSC+OEC co-transplantation may promote axonal regeneration and the recovery of nerve function.

However, how can functional nerve and axon regeneration be improved? Research to date indicates the importance of NTFs, particularly BDNF and NGF, during recovery after PNI (Sendtner et al., 1992; Zhang et al., 2000; Shakhbazau et al., 2012a; Tang et al., 2013). A growing amount of evidence indicates that, in addition to providing structural support for growing axons by the expression of NTFs (Stoll and Müller, 1999), OECs release many NTFs, including NGF, BDNF and GDNF (Bunge et al., 1989; Brown et al., 1991). NGF is known to guide axons (Yu et al., 2010), promote axonal sprouting (Tuszynski et al., 1996), and cell migration (Cao et al., 2007; Figure 3G, 8N), stimulate myelination (Chan et al., 2004), improve the regeneration of sensory neurons and reduce denervated muscle atrophy (Crowley et al., 1994; Shakhbazau et al., 2012a; Figure 7L, 8N), and eventually to improve functional recovery after injury (Röyttä et al., 1999; Kemp et al., 2011). In our study, the expression of NGF in the EPI-NCSC+OEC group was higher than in the other group (Figure 8G–L,N), indicating that NGF might participate in nerve repair. Up-regulation of NGF might also play a vital role in nerve repair by increasing the number of myelin sheathes, which was greater in the EPI-NCSC+OEC group than in the DMEM/F_12_, EPI-NCSC or OEC groups (Figure 6F). Besides, BDNF is important for stimulating axonal elongation (Acheson et al., 1991; Zhang et al., 2000; English et al., 2005; Wilhelm et al., 2012) and for survival of motoneurons (Sendtner et al., 1992; Yan et al., 1992; Koliatsos et al., 1993). We also found that the expression of BDNF in the EPI-NCSC+OEC group was greater than in the other group (Figure 8A–F,M). Likewise, it reported that OEC-M treatment after contusive SCI increased BDNF levels and then improved the function recovery and promoted the axonal regeneration (Pastrana et al., 2007; Gu et al., 2017). Herein, high expression level of BDNF and NGF in the EPI-NCSC+OEC group might improve nerve function and promote the axonal regeneration. Therefore, in this study we speculated that the mechanism that EPI-NCSC+OEC co-transplantation repaired PNI might be up-regulation of BDNF and NGF, but BDNF and NGF might derive from: (1) OEC might secrete, which have been reported to express BDNF and NGF and facilitate axonal regeneration after SCI (Lipson et al., 2003); (2) Schwann cells might secrete BDNF and NGF after PNI *in vivo* (Shakhbazau et al., 2012b); (3) EPI-NCSC might secrete little BDNF and NGF (Sieber-Blum et al., 2006), but the combination of EPI-NCSC and OEC might heighten the expression level of BDNF and NGF, which have been reported that co-culture of Schwann cells and adult stem cells led to synergistic neurotrophic effects (BDNG and NGF) in PNI (Dai et al., 2013) and synergic effects of EPI-NCSCs and OECs increased the expression of BDNF and GDNF in SCI (Zhang et al., 2015). One maybe that the combination of cells promote the secretion of BDNF and NGF from Schwann cells of donor; another maybe that EPI-NCSC prompts the expression of BDNF and NGF from OECs. However, specific origin of BDNF and NGF remain to further investigate.

Overall, our results unveiled a beneficial effect of co-transplantation of EPI-NCSCs and OECs after PNI, whereas many issues remained to be determined such as the differentiation of stem cells *in vivo*. EPI-NCSC and OEC co-transplantation was a promising and easily transformable approach that could lead to significant amelioration of patients suffering from PNI.

## Conclusion

Our findings indicated that EPI-NCSC and OEC co-transplantation promoted sciatic nerve regeneration and improves nerve function. Moreover, the mechanism of PNI improved by EPI-NCSC and OEC co-transplantation was likely to up-regulate the expression of BDNF and NGF. The application of EPI-NCSC and OEC co-transplantation in clinical trials might improve clinical outcomes and provided a new methods for PNI.

## Data Availability

The raw data supporting the conclusions of this manuscript will be made available by the authors, without undue reservation, to any qualified researcher.

## Ethics Statement

All experimental procedures with animals were approved by the local institution review board and were carried out according to the guidelines of the Third Military Medical University (Chongqing, China) for the care and use of laboratory animals.

## Author Contributions

LZ performed the experiment, conceived the study design, analyzed the data, and drafted the manuscript. BLi and BLiu participated in conception and design of the experiments. ZD modified the manuscript.

## Conflict of Interest Statement

The authors declare that the research was conducted in the absence of any commercial or financial relationships that could be construed as a potential conflict of interest.

## Abbreviations

BDNF: brain derived growth factor
cMAPs: compound muscle action potentials
DMEM/F_12_: dulbecco’s modified eagle medium: nutrient mixture F-12
ECM: extracellular matrix
ELISA: enzyme-linked immuno sorbent assay
EPI-NCSCs: epidermal neural crest stem cells
GDNF: glial cell line-derived neurotrophic factor
H&E: hematoxylin and eosin
IHC: immunohistochemistry
NCV: nerve conductive velocity
NGF: nerve growth factor
NTE: nerve tissue engineering
NTFs: neurotrophic factors
OECs: olfactory ensheathing cells
PNI: peripheral nerve injury
PNS: peripheral nerve system
SC or SCL_4-6_: lumbar spinal cord at L4–L6
SCI: spinal cord injury
SFI: sciatic function index
SN: sciatic nerve
TEM: transmission electron microscopy
