# Supplementary material for: Co-transplantation of Epidermal Neural Crest Stem Cells and Olfactory Ensheathing Cells Repairs Sciatic Nerve Defects in Rats
Source: Front Cell Neurosci. 2019 Jun 12;13:253. doi: 10.3389/fncel.2019.00253 (PMC6582070; doi:10.3389/fncel.2019.00253)
Supplement: Supplementary file 1 [file Table_1.doc]

**The isolation and culture of EPI-NCSCs**

Briefly, Whisker pads of GFP-adult Sprague Dawley rat was cut under anaesthetizing for getting the complete hair follicle, then the connective tissues were carefully removed, and hair follicle external root sheath with juga intact was put in the culture dish coated by rat tail collagen. After 30 min, 1.5 mL culture medium (10% FBS, 2% B27, bFGF 20 ng/mL, 5% day-11 chick embryo extract, 1% penicillin/streptomycin) (GIBCO, USA) was added for cell culture at 37℃ in a 5% CO_2_ incubator. On the third day, half of the culture medium was changed, on the sixth day, cell overspread around the whole hair follicles, followed by removing hair follicles, then digested by 0.25% trypsin/0.02% EDTA (GIBCO, USA) to subculture. The expansion culture medium containing 90% DMEM/F_12_, 10% day-11 chick embryo extract, stem cell factor (5 ng/mL), bFGF-2 ( 20 ng/mL), neurotrophin-3 (10 ng/mL), epidermal growth factor (20 ng/mL), and ITS+3 (insulin, transferrin, selenium, and three essential fatty acids) was changed every three days.

**The isolation and culture of OECs**

Briefly, olfactory bulb was separated, then the caudal one-third of the bulb was removed and discarded along with as much white matter as possible to isolate the outer nerve layer. Tissue was finely minced with microscissors, digested with 0.25% trypsin/0.02% EDTA (GIBCO, USA), incubated for 30 min at 37°C in 5% CO_2_ incubator. The digestion was stopped by adding 20% FBS (GIBCO, USA), and gently blew digestion solution with firepolished pasture pipettes so as to get free cells. The cell suspension was resuspended, and centrifuged. Cell suspension is transferred to culture dish with culture medium (10% FBS, Forskolin 2 µM/mL, bFGF 10 ng/mL, 1% penicillin/streptomycin) (GIBCO, USA). On the third day, half of the culture medium was changed. Subsequently, the culture medium was changed every three days.

**The identification of EPI-NCSCs, OECs**

Cells were washed with phosphate buffer (PBS) for 3 times, fixed with 4% Polyoxymethylene (PFA) for 30 min at room temperature (RT) and permeated with 0.3% Triton X-100 for 30 min. Then washed with PBS for 3 times (5 min each time). Blocked with 1% BSA for 30 min at RT, incubated with primary antibody mouse anti-Nestin (1:100, Abcam, USA), rabbit anti-SOX10 (1:1000, Abcam, USA), rabbit anti p75 (1:1000, abcam, USA) and mouse anti S-100 (1:500, abcam, USA) overnight at 4℃. Washed with PBS for 3 times (5 min each time), then incubated with second antibody Alexa Flour 594 goat anti-rabbit IgG (1:50, Beyotime, China), Cy5 goat anti-mouse IgG (1:50, Beyotime, China), Alexiflour 488 goat anti-mouse(1:1000, abcam, USA), and Alexiflour 594 goat anti-rabbit (1:1000, abcam, USA). Washed with PBS for 3 times (5min each time), and counterstained with Hoechst33342 (1:1000, sigma, USA) 1h at RT. Washed with PBS for 3 times (10 min each time), dried at RT and covered with Fluoromount anti-fade reagent (Sigma, USA). Samples were observed under fluorescence microscope.

**The fabrication and degradation of nerve conduits**

The nerve conduits were fabricated from 10% PLGA (Sigma, USA) dissolved in CHCl_3_ with a mold. Dried for 48 h and removed from the mold and cut into 15 mm length, and lyophilized in vacuum freeze dryer for 24 h. Sterilized by gamma irradiation for 30 min, and conserved until use.

The nerve conduits were in heat-sealed pouches made from 10 µm nylon mesh and vacuum-dried for 24 h to obtain tare weights. Samples were then put in 50 mL 0.01M PBS (pH 7.4) and incubated at 37℃, 5% CO_2_ incubator. Replaced incubation buffer with new buffer, and monitored pH weekly. At regular intervals, for a total of 30 weeks, samples were taken out, washed repeatedly in ultrapure water, and vacuum-dried for 24h. Dried pouches were put in 5 mL tetrahydrofuran (THF) and shaken overnight to dissolve the nerve conduits. Empty pouches were taken out, washed repeatedly in CHCl_3_, vacuum-dried overnight, and weighed.

Degradation was characterized by mass loss and pH change. Mass loss was determined by weighing dried samples at given time point (m_t_) and compared to the initial mass(m_o_):

Mass loss(%) =(1－m_t_/m_o_)×100%

**Animal model and transplantation**

The nerve conduits were immersed in DMEM/F_12_ for 1h at 37℃ before transplantation. Cells were resuspended in DMEM/F_12_ (4-5×10^4^ cells/µL). The cell suspension was mixed with extracellular matrix (ECM) (sigma, USA) at a 1:1 ratio (volume ratio), and injected into the nerve conduits (50 µL). The volume of single EPI-NCSCs or OECs was 25 µL. The volume of the mix was 25 µL, in which the number of EPI-NCSCs and OECs respectively was equal to single EPI-NCSCs or OECs.

The establishment of model: the right sciatic nerve was severed with a microscissors and 5 mm length nerve was cut off. The nerve conduit (15 mm in length) was bridged between nerve proximal end and distal end, which was sutured under the microscope with 8-0 sutures, generating a gap of 10 mm nerve defect model.

**Histological observation and survival of transplanted cells *in vivo***

The rats were anesthetized with 3% pentobarbital sodium (70 mg/kg) (Sigma, USA), transcardially perfused with 0.01M PBS, and followed with 4% PFA eight weeks after transplantation. Samples were obtained, fixed with 4% PFA and then dehyarated with 30% sucrose overnight. Samples were cut into a thickness of 20 µm sections by freezing micritome (Lecia CM1520, Germany) for H&E staining. The sections were observed under fluorescent microscope and the pictures were acquired by fluorescent camera (DP74, Olympus, Japan). To obtain a semi-quantitative assessment of cell survival, five serial longitudinal sections along the central axis were cut. The number of cells of each section was counted by Image J. We calculated cell number per sample (area, 100 µm × 100 µm, 20 µm thickness), and total cell number of sample (area, 100 µm × 100 µm, 500 µm thickness). Cell counting method: we used ImagJ software and counted cell number. the procedure of ImagJ software: open picture → Image → 8-bit → Edit → Invert → Image → Adjust → Threshold → Analyze → Analyaz Particales → click OK.

**Retrograde tracing**

Eight weeks after transplantation, the sciatic nerve was exposed under anesthetization, and 2% DiI solution (15µL) (Sigma, USA) was injected into the nerve trunk 10mm proximal to the suturing point with a microinjector. After injection, the needle was kept in situ for 5 min. The incision was sutured. After 24 h, rats were anesthetized with 3% pentobarbital sodium (70 mg/kg) (Sigma, USA) and transcardially perfused with 0.01M PBS, and followed with 4% PFA. The lumbar spinal cord at L4–L6 (SCL_4-6_) was harvested, fixed with 4% PFA, dehydrated with 30% sucrose, and sectioned transversely into 15 µm sections. Neurons labeled with DiI (red) were observed by fluorescent microscope (BX 51WI, Olympus, Japan). DiI-labeled motoneurons were quantified by Image J.

**Evaluation of nerve fiber morphology and cell apoptosis**

Eight weeks after transplantation, the graft was harvested, processed, and stained by toluidine blue. Briefly, samples were fixed, embedded with resin, sectioned into 2 µm thickness with a microtome, and stained with 1% toluidine blue. Another portion of samples was cut into ultrathin sections to stain with lead citrate and uranyl acetate, and followed by observation under transmission electron microscope. Choose five pictures per sample, calculate the mean, and analyze data. The density and thickness of myelinated nerve fibers were quantified by ImageJ software.

**TUNEL staining**

Samples were harvested, fixed with 4% PFA, dehydrated with 30% sucrose, and sectioned into 15 µm sections. TUNEL staining was carried out by In Situ Cell Death Detection Kit (Roche, Germany) according to the manufacturer’s instructions. Apoptotic neurons were quantified by ImageJ software.

**SFI**

Briefly, the hind paws were dipped on the ink and the animals walked on white paper leaving five to six prints. Rats were acclimatized experiments before surgery. Foot prints from the normal (N, right side) and experimental (E, left side) were collected transplantation after 2, 4, and 8 weeks. Foot prints were measured for the following parameters: the entire plantar length (PL): the distance from the first to fifth toes; the toe spread (TS): the distance between the second and fourth toes; the intermediary toe spread (IT). The SFI was calculated according to the following formula:

**SFI=-38.3[(EPL-NPL)/NPL]+109.5[(ETS-NTS)/NTS]+13.3[(EIT-NIT)/NIT]-8.8**

SFI ranged from -100 to 0. The value 0 describes normal function and the value -100 shows complete transaction of sciatic nerve.

**Analysis the expression of BDNF and NGF**

BDNF and NGF play a vital role in repairing PNI, therefore we assess the expression of BDNF and NGF by IHC and ELISA after transplantation. The rats were anesthetized with 3% pentobarbital sodium (70 mg/kg) (Sigma, USA). The SCL_4-6_ were harvested, fixed with 4% PFA, dehydrated with 30% sucrose, embedded with tissuse freezing medium (OCT, Japan), and sectioned into 15 µm sections. We used primary antibody ribbit anti-BDNF (1:1000, SANTA Cruz, USA) and ribbit anti NGF (1:1000, Abcam, USA) to analysis the expression of BDNF and NGF. The secondary antibodies were Alexa Fluor 647 goat anti-ribbit IgG (1:500, Abcam, USA) in 1% Triton/PBS. Pictures were obtained by the fluorescent camera (DP74, Olympus, Japan). IHC analysis were quantified by ImageJ software. Get three samples, choose five pictures per sample, count BDNF positive cells per µm^2^, multiply by 10^4^, and calculate the mean. Similarly, calculate NGF positive cells in this way.

Eight weeks after transplantation, the expression of BDNF and NGF was quantified by ELISA. Samples were harvested, rapidly frozen in liquid nitrogen, and stored at -80℃ until further use. Briefly, samples were taken out from liquid nitrogen and finely dissected. Resuspended in 500 µL of NP-40 extraction buffer containing 150 mM NaCl, 50 mM Tris-HCl (pH 8.0), 0.1% Tween-20, 1% NP-40 and protease inhibitor PMSF, further homogenized with a voeter mixer for 30 s, 12000 r/min centrifuged for 30 min at 4℃, reserved supernatant, and measured the concentration of total protein by BCA assay. The concentration of NGF and BDNF was measured by ELISA kit (Abcam, USA) according to the manufacturer’s instructions.
